# Gray hair influences perceived age and social perceptions

**DOI:** 10.3389/fpsyg.2025.1541836

**Published:** 2025-05-07

**Authors:** Kallye M. Nutt, Christopher A. Thorstenson, Jessica L. Yorzinski

**Affiliations:** ^1^Department of Biology, Texas A&M University, College Station, TX, United States; ^2^Munsell Color Science Laboratory, Rochester Institute of Technology, Rochester, NY, United States; ^3^Department of Ecology and Conservation Biology, Texas A&M University, College Station, TX, United States

**Keywords:** ageism, aggressiveness, attractiveness, gray hair, mate choice, sexual selection, social status, trustworthiness

## Abstract

Physical traits can influence how people are perceived and evaluated by others, often reflecting underlying qualities considered important for social interaction. Gray hair color is one such trait that can potentially alter social perceptions related to aging, but has rarely been investigated independently from other correlated physical characteristics. The aim of the current work is to investigate how gray hair independently influences important social evaluations including perceived age, attractiveness, social status, aggressiveness, and trustworthiness. Participants (*N* = 120) were presented with images of male and female faces that exhibited non-gray hair (brown, blonde or red), and versions of the same faces manipulated to have gray hair, and were asked to rate these faces according to those social evaluations. Linear mixed-effects models indicated that faces with gray hair were perceived as older and less attractive. Men (but not women) also perceived faces with gray hair as less trustworthy. Results showed that gray hair did not impact assessments of social status or aggression. These results suggest that gray hair is independently used as an indicator of some important social evaluations (age, attractiveness, and trustworthiness), while others (social status and aggression) may be better informed by other characteristics.

## 1 Introduction

People readily categorize others into groups, an adaptive process allowing us to rapidly organize our complex social environment (Macrae et al., 1994). Perceptions of age are among the first categorizations that are formed when encountering someone (Cuddy and Fiske, 2002; Fiske, 1998). People perceived as being older are often characterized as having high warmth (e.g., friendly, good-natured, sincere and warm) but low competence (e.g., capable, competent, confident, and skillful; Fiske et al., 2002). Perceptions of age can be predicted by physical traits. For example, wrinkles, age spots, arcus senilis and pattern baldness tend to be perceived as indictors of older age (Bulpitt et al., 2001; Gunn et al., 2009). Gray hair is another physical trait that is often associated with aging (Bulpitt et al., 2001; Gunn et al., 2009).

Gray hair becomes more prevalent with increasing age (Bulpitt et al., 2001; Gunn et al., 2009; Keogh and Walsh, 1965; Panhard et al., 2012), with a significant increase in gray hair between the ages of 45 and 60 years old (O’Sullivan et al., 2020; Panhard et al., 2012). Similar to other physiological changes associated with aging that involve declines in the ability of tissues to maintain homeostasis and regenerate new tissue, gray hair develops when melanocytes cannot maintain homeostasis and replenish melanin (the pigment that colors new hair; Nishimura, 2011; Sarin and Artandi, 2007). Gray hair may therefore be an honest indicator of biological aging (Jawor and Breitwisch, 2003). It is also possible for hair to turn gray in younger people; such premature graying of hair may result from genetic predispositions, environmental factors, or psychological stress (Pandhi and Khanna, 2013).

From an evolutionary perspective on human mate selection (Buss, 1996), men generally seek women with high reproductive potential, a status that is reinforced by qualities including youth and health. Conversely, women tend to seek men who have high social status. These qualities are associated with aging, as reproductive potential in women and social status in men declines with advanced age (Conroy-Beam and Buss, 2019). Therefore, observable characteristics associated with aging likely convey social information that influences inferences related to mate quality and competition (Buss and Schmitt, 2019), even when mate seeking is not an imminent goal. Such age-related signals may be conveyed indirectly through observable characteristics like hair color (Sorokowski, 2008; Fink et al., 2016). Because decreased hair quality, including graying, is linked to aging (Bulpitt et al., 2001), it can be inferred as a signal for reduced reproductive capability in women (Hinsz et al., 2001), decreased health status (Mesko and Bereczkei, 2004), and poorer social status in men. Thus, these inferences are likely to be reflected in evaluations of perceived age, but also in perceptions related to age, such as attractiveness, social status, aggressiveness, and trustworthiness.

Hair color has indeed been shown to influence social perceptions (Aberg et al., 2020; Barrett, 2003; Beddow, 2011; Feinman and Gill, 1978; Kyle and Mahler, 1996; Swami and Barrett, 2011). Both men and women are perceived as being older when they have more gray hair (Bulpitt et al., 2001; Gunn et al., 2009). Based on questionnaires and qualitative reports, women perceive gray hair as unattractive and associated with poor health and social disengagement (Clarke and Korotchenko, 2010; Ward and Holland, 2010). Several studies have suggested that men and women could potentially leverage their gray hair as a sign of credibility and competence (Isopahkala-Bouret, 2016; Mitra et al., 2014; Ward and Holland, 2010). While the above studies provide important insight into the effects of gray hair, we are unaware of any experimental studies that have specifically examined the effects of gray hair on perceived age and social perceptions, independently of other correlated qualities.

### 1.1 Current research

We therefore aimed to experimentally examine the influence of gray hair on perceived age and social perceptions. Because gray hair is more common with older age (Keogh and Walsh, 1965; Panhard et al., 2012), we predicted that men and women with gray hair would be perceived as older. Previous research has demonstrated that men and women consider older faces to be less attractive (Foos and Clark, 2011; He et al., 2021; Kwart et al., 2012) and less trustworthy (Pehlivanoglu et al., 2022; Zebrowitz et al., 2013; but see Fiske et al., 2002) than younger faces. In addition, male faces that are older are considered less aggressive (Richardson et al., 2020) and have lower social status (plateauing at approximately 35 years old; Batres et al., 2015; Mueller and Mazur, 1996; Roll and Verinis, 1971). Because no previous studies have experimentally assessed whether gray hair impacts social perceptions, we decided to focus our study on social perceptions that are commonly explored in face perception studies: attractiveness, social status, aggressiveness, and trustworthiness (e.g., Carré et al., 2009; Goetz et al., 2013; Jones et al., 2021; Little et al., 2011; Stirrat and Perrett, 2010; Sutherland et al., 2013). Assuming gray hair is an indicator of older age, we predicted that faces with gray hair would be rated as less attractive, less aggressive (men only), lower social status (men only), and less trustworthy, than those with non-gray hair. We tested these predictions by presenting men and women with photographs of faces exhibiting non-gray (brown, blonde or red) or gray hair. The subjects evaluated these photographs based on perceived age as well as other related social perceptions (attractiveness, social status, aggression, and trustworthiness).

## 2 Materials and methods

### 2.1 Participants

One hundred and twenty (60 men and 60 women) people participated in this study at Texas A&M University from September 2022 through December 2023. They were between the ages of 18 and 43 years old (mean ± SE: 23 ± 0.4 years old). 97 and 93% of the men and women participants, respectively, were 30 years old or younger. Most of the participants (80%) indicated that they were heterosexual. The self-reported ethnic group of the participants was Caucasian (36%), Asian (36%), Hispanic (12%), multi-racial (6%), African American (6%), and unspecified (5%). Most of the female participants (73%) had not taken hormonal contraceptives within 3 months prior to participating. We recruited the participants through emails. The participants were told that they would be participating in a study that explored face perception and they each earned $15.

### 2.2 Experimental stimuli and procedure

**Figure 1:**
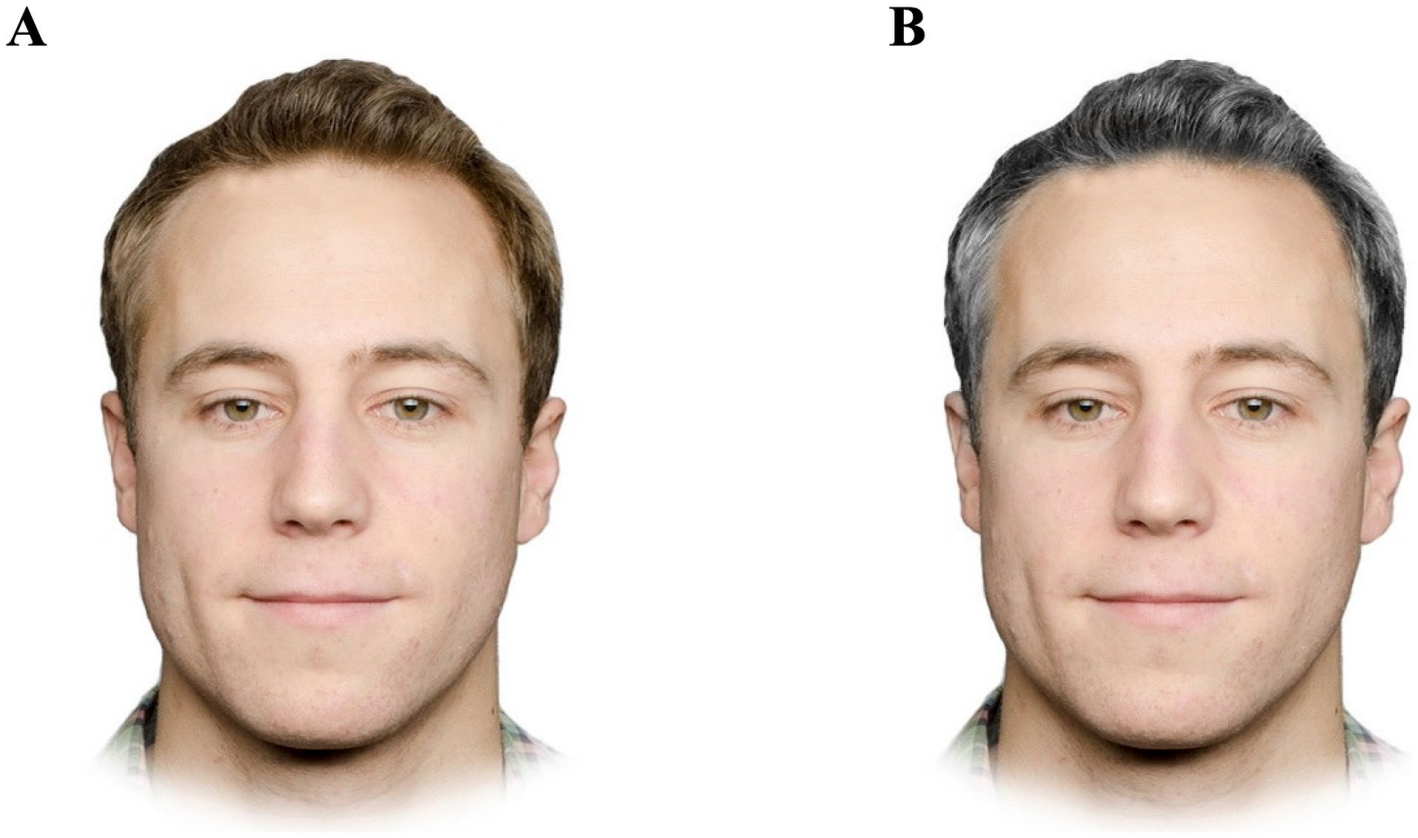
Example of face stimuli displaying a male with **(A)** non-gray and **(B)** gray hair color. The man in the stimuli gave informed consent for his photographs to be used for scientific purposes (Chelnokova et al., 2014, reproduced with permission from the Oslo Face Database, https://affectivebrains.com/oslo-face-database/). Face stimuli displaying a woman are not shown due to lack of consent for their images being used as examples in publications.

The experimental stimuli consisted of photographs of men and women that exhibited non-gray hair (blonde, brown, or red) and gray hair. The stimuli of people with non-gray hair were selected from a face database (Chelnokova et al., 2014; Oslo Face Database) and we obtained permission to use these stimuli in our study. The face database included photographs of students from the University of Oslo (Oslo, Norway). We created the stimuli of people with gray hair by modifying the stimuli with non-gray hair. We did so by creating a color mask using Matlab (Matlab, 2022) and Photoshop (Adobe Systems, USA), so that color manipulations were constrained only to hair on the head while keeping color unchanged across the rest of the image. In Matlab, we converted the sRGB image into CIELab color space and then multiplied the lightness of each image (L*) by a scalar (1.2) while setting the other color variables (a* and b*) to zero (removing the chromatic content of the hair). For images in which the original hair color was very light, we first subtracted 10 units from L* before applying the scalar so that the hair color would not appear ‘washed out’; conversely, for images in which the original hair color was very dark, we first added 5 or 10 units to L* before applying the scalar so that the hair color was not too dark. This step of adding or subtracting units of L* was determined subjectively but resulted in hair that we qualitatively assessed as appearing naturally gray. This process resulted in two versions of the same face: one in which the hair color was the original (non-gray) color and one in which the hair color was gray (Figure 1). The final stimuli consisted of 140 faces (faces of 35 men and 35 women with non-gray hair, and faces of the same people with gray hair) and a summary of their hair colors is included in Table 1.

**Table 1:** Summary of hair colors in the experimental stimuli.

Stimuli gender	Hair color	L*	a*	b*
Male	Non-gray	27 12–48	5 2–8	13 5–23
	Gray	35 23–46	0 –	0 –
Female	Non-gray	36 18–58	5 3–10	17 9–27
	Gray	41 26–57	0 –	0 –

The colors are in the CIELab color space, with L* representing lightness, a* representing the red-green component, and b* representing the yellow-blue component (Fairchild, 2013). Means and ranges are displayed.

The subjects viewed the stimuli on a computer monitor (EIZO FlexScan EV2451) while resting their chins in a chin cup (UHCOTech HeadSpot). Their gaze was monitored with an eye-tracker (Tobii Pro Spectrum) but those data are not reported here. We used custom Matlab scripts to program the experiments (Niehorster et al., 2020). The monitor was color calibrated at least once every 2 weeks (Calibrite ColorChecker Display Pro; luminance: 120 cd/m^2^; gamma = 2.2; white point CCT = 6,500 K). Each subject evaluated all of the face stimuli (*n* = 140) twice: they assessed the faces based on social perceptions and perceived age. The social perceptions included evaluations of attractiveness, social status, aggressiveness, or trustworthiness. Social status was defined as how likely the person in the image was to have a high-ranking social position and command respect over other people in the community (Dixson and Vasey, 2012). A given subject only evaluated the stimuli based on one of the four social perceptions. The order of the evaluations (social perception or perceived age) was randomized across subjects and the subjects took a two-minute break between them. Thirty subjects (15 men and 15 women) participated in each of the four social perception experiments (attractiveness, social status, aggressiveness, or trustworthiness) for a total of 120 subjects. The sample size was similar to other studies on social perception (Yorzinski and Platt, 2010; Fink et al., 2016). The same experimenter (K.N.) collected all of the data.

Each face stimulus was displayed for 5 s (the same duration used in other studies of social perception; e.g., Bowdring et al., 2021; Walla et al., 2020; Yorzinski and Platt, 2010) and then an assessment screen appeared (the face was not visible during the assessment screen). The subjects indicated their assessment of the preceding face (via a mouse click) before viewing the next face. The social perception assessment was scored on a scale of 1 to 10, with text above the numbers to remind subjects of the scale. Depending on the experiment, above the number one, it stated “least attractive,” “lowest social status,” “least aggressive,” or “least trustworthy;” above the number 10, it stated “most attractive,” “highest social status,” “most aggressive,” or “most trustworthy.” The perceived age assessment options ranged from 18 to 77 years old. In rare cases (45/33,600 stimuli; 0.13%), the subject did not click on a social perception or age score for a stimulus (i.e., they clicked on a blank spot within the assessment screen) and the data for such stimuli were therefore not included in the analysis. The face stimuli were displayed in blocks of male or female faces; block order was randomized across subjects. The face stimuli within each block were also randomized across subjects except that the faces of the same individuals (with different hair colors) were never shown consecutively. In summary, each subject evaluated the faces of 35 women and 35 men (both the gray hair and non-gray hair versions of each face, for a total of 140 faces) based on social perceptions (attractiveness, social status, aggressiveness, or trustworthiness) and perceived age. After finishing the experiment, each subject competed the Aging Perceptions Questionnaire (APQ; Barker et al., 2007). The APQ evaluates aging perceptions along seven domains. An overall score on the APQ is calculated by summing the scores across all domains (after reverse scoring ‘Consequences Positive’ and ‘Control Positive’; Fawsitt et al., 2022), with higher scores indicating more negative perceptions of aging.

### 2.3 Measurements and statistical analysis

We analyzed our assessment data using linear mixed-effects models with repeated measures in SAS (PROC MIXED; Version 9.4; SAS Institute Inc.). The dependent variables were the perceived age or social perception scores. The independent variables were the hair color of the people in the stimuli (‘stimuli hair color’: non-gray or gray), gender of the face stimuli (‘stimuli gender’), gender of the subject (‘subject gender’), and their interactions as well as the subjects’ overall score on the APQ (‘perception of aging’), the interaction between their overall score on the APQ and hair color of the people in the stimuli, and subject age. Subject identity was included within the models to account for repeated measures. Stimuli identity was also included within the models to account for repeated measures because the models used the raw scores from each subject for each stimuli rather than averages. We also performed four comparisons within each model of social perception to compare the influence of gray hair on both face stimuli and subject gender; we used a Bonferroni correction to evaluate statistical significance. Raw data are included in the Supplementary material. We also reran the above statistical models on a dataset that excluded short and long reaction times (we excluded any stimuli in which the subject took less than 1.01 s or more than 4.27 s to indicate their score, which represent the lower and upper 5% of the latency data); the results were qualitatively the same as the full dataset and are reported in the Supplementary Tables S1, S2.

## 3 Results

**Figure 2:**
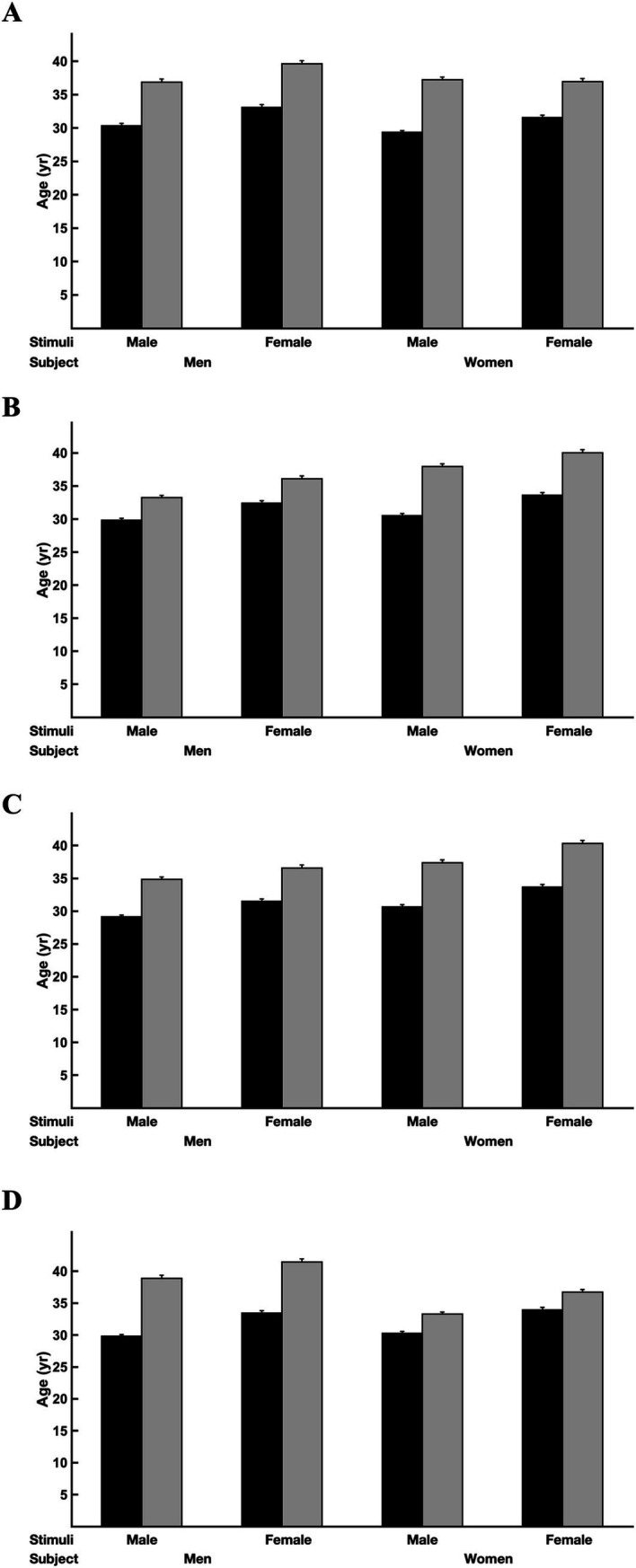
The effect of stimuli hair color on perceived age in the **(A)** attractiveness, **(B)** social status, **(C)** aggression, and **(D)** trustworthiness experiments based on the gender of the face stimuli and subjects.

**Figure 3:**
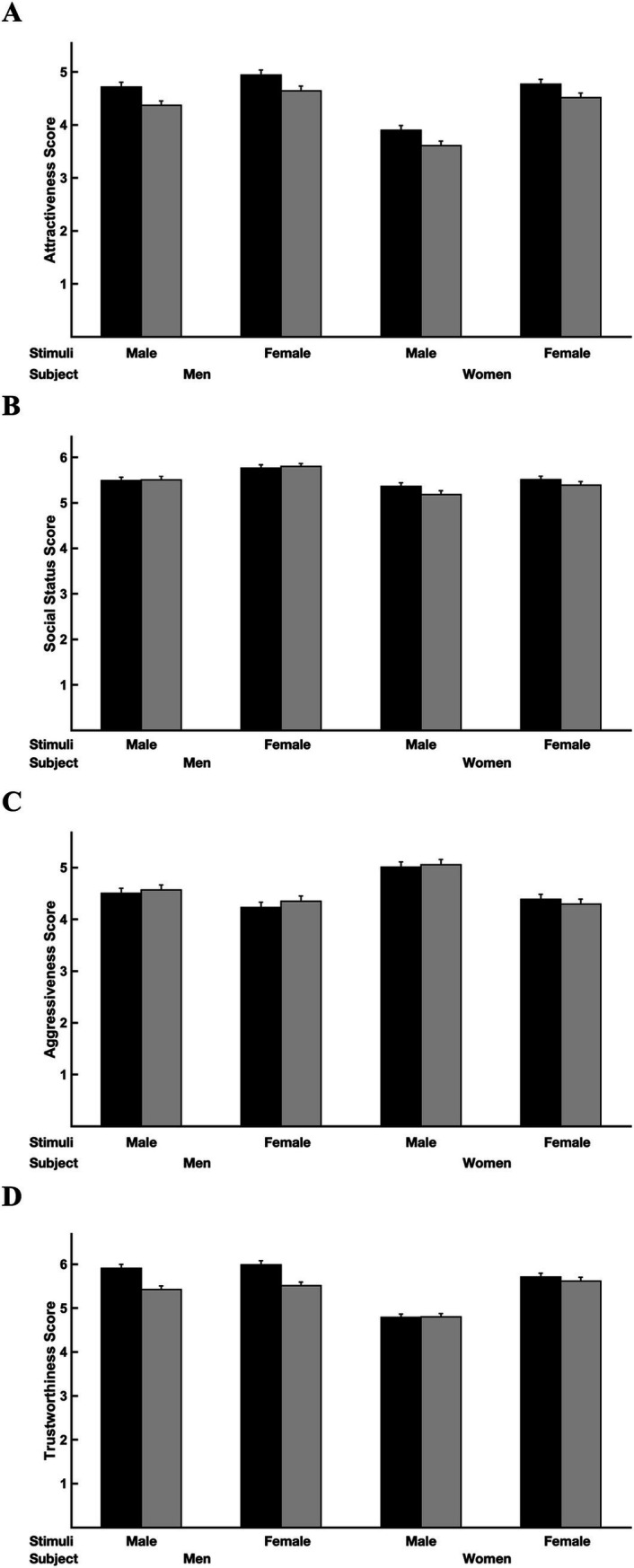
The effect of stimuli hair color on **(A)** attractiveness, **(B)** social status, **(C)** aggression, and **(D)** trustworthiness based on the gender of the face stimuli and subjects.

Gray hair color increased the perceived age of male and female faces (*p* < 0.001; Table 2 and Figure 2). Faces with gray hair were, on average, evaluated as being 1.2× older than faces without gray hair. For example, women assessed the perceived ages of the male faces with non-gray hair as 30 years old (SE: 0.15 years), on average, and assessed the perceived ages of the male faces with gray hair as 36 years old (SE: 0.18 years); men assessed the perceived ages of the female faces with non-gray hair as 33 years old (SE: 0.19 years), on average, and assessed the perceived ages of the female faces with gray hair as 38 years old (SE: 0.24 years). Gray hair color decreased the attractiveness of male and female faces (*F*_1,28_ = 4.73, *p* = 0.038) but had no influence on their social status (*F*_1,28_ = 0.70, *p* = 0.41) or aggressiveness (*F*_1,28_ = 1.21, *p* = 0.28). Gray hair also decreased the trustworthiness of faces when evaluated by men (male faces: *t*_1,28_ = 4.48, *p* = 0.0001; female faces: *t*_1,28_ = 4.50, *p* = 0.0001); gray hair had no impact on the trustworthiness of faces evaluated by women (*p* > 0.52; Table 3 and Figure 3). Means, standard deviations, 95% confidence intervals, and effect sizes are reported in Table 4.

**Table 2:** The effect of stimuli hair color, stimuli gender, subject gender, perception of aging, and subject age on perceived age in each experiment.

Independent variables	Attractiveness: age	Social status: age	Aggression: age	Trustworthiness: age
Stimuli hair color	71.02 (<0.0001)*	22.29 (<0.0001)*	14.52 (0.0007)*	22.01 (<0.0001)*
Stimuli gender	54.66 (<0.0001)*	118.59 (<0.0001)*	113.68 (<0.0001)*	181.19 (<0.0001)*
Subject gender	0.52 (0.48)	2.50 (0.13)	1.12 (0.30)	1.57 (0.22)
Stimuli gender × stimuli hair color	6.58 (0.016)*	0.56 (0.46)	0.48 (0.50)	1.69 (0.20)
Stimuli gender × subject gender	12.51 (0.0014)*	0.050 (0.82)	4.03 (0.054)	0.92 (0.35)
Stimuli hair color × subject gender	0.30 (0.59)	48.07 (<0.0001)*	7.26 (0.012)*	125.39 (<0.0001)*
Stimuli hair color × stimuli gender × subject gender	6.07 (0.02)*	1.75 (0.20)	0.28 (0.60)	0.63 (0.43)
Perception of aging	0.27 (0.60)	2.90 (0.10)	0.20 (0.66)	0.37 (0.55)
Perception of aging × stimuli hair color	15.07 (0.0001)*	1.26 (0.26)	0.020 (0.89)	0.66 (0.42)
Subject age	0.020 (0.88)	0.68 (0.42)	0.00 (0.96)	0.030 (0.86)

*F values with p-values in parentheses are displayed. The numerator degrees of freedom is 1 and the denominator degrees of freedom is 28. Statistically significant results are indicated with an asterisk.*

**Table 3:** The effect of stimuli hair color, stimuli gender, subject gender, perception of aging, and subject age on social perceptions.

Overall model	Attractiveness	Social status	Aggression	Trustworthiness
Independent variables				
Stimuli hair color	4.73 (0.038)*	0.70 (0.41)	1.21 (0.28)	3.11 (0.089)
Stimuli gender	125.58 (<0.0001)*	21.98 (<0.0001)*	65.26 (<0.0001)*	72.60 (<0.0001)*
Subject gender	0.46 (0.51)	0.26 (0.61)	0.42 (0.52)	1.99 (0.17)
Stimuli hair color × stimuli gender	0.13 (0.72)	0.16 (0.70)	0.15 (0.70)	0.22 (0.64)
Stimuli gender × subject gender	40.16 (<0.0001)*	1.23 (0.28)	14.83 (0.0006)*	49.76 (<0.0001)*
Stimuli hair color × subject gender	0.51 (0.48)	3.28 (0.081)	0.69 (0.41)	18.23 (0.0002)*
Stimuli hair color × stimuli gender × subject gender	0.00 (0.98)	0.05 (0.82)	0.72 (0.40)	0.20 (0.65)
Perception of aging	3.55 (0.071)	4.70 (0.040)*	1.43 (0.24)	0.040 (0.85)
Perception of aging × stimuli Hair color	1.35 (0.25)	0.40 (0.53)	1.43 (0.23)	6.73 (0.0095)*
Subject age	0.30 (0.59)	5.71 (0.024)*	0.00 (0.98)	0.07 (0.79)

Comparisons			Attractiveness	Social status	Aggression	Trustworthiness
Subject gender	Stimuli gender	Stimuli hair color				
Men	Men	Natural vs. gray	3.51 (0.0015)*	0.18 (0.86)	0.48 (0.64)	4.48 (0.0001)*
	Women	Natural vs. gray	3.11 (0.0042)*	0.35 (0.73)	0.94 (0.35)	4.50 (0.0001)*
Women	Men	Natural vs. gray	2.75 (0.010)*	1.86 (0.074)	0.5 (0.62)	0.26 (0.79)
	Women	Natural vs. gray	2.42 (0.022)*	1.24 (0.23)	0.74 (0.47)	0.65 (0.52)

For the overall model, *F* values with *p*-values in parentheses are displayed; for the comparisons, *t* values with *p*-values in parentheses are displayed. Statistically significant results are indicated with an asterisk.

**Table 4:** The mean, standard deviation, 95% confidence interval, and effect size (Cohen’s *d*) of non-gray hair versus gray hair on assessments of perceived age and social perceptions.

Assessment	Experiment	Subject gender	Stimuli gender	Non-gray hair			Gray hair			Cohen’s *d*
				Mean	SD	95% CI	Mean	SD	95% CI	
Perceived age	Attractiveness	Men	Male	30.35	7.65	29.69–31.01	36.89	10.34	36–37.77	0.72
			Female	33.09	9.37	32.29–33.89	39.60	11.29	38.63–40.57	0.63
		Women	Male	29.35	6.19	28.82–29.88	37.24	9.25	36.45–38.03	1.00
			Female	31.56	8.42	30.84–32.28	36.93	11.24	35.96–37.89	0.54
	Social Status	Men	Male	29.83	6.31	29.29–30.37	33.26	7.38	32.63–33.89	0.50
			Female	32.40	8.77	31.65–33.15	36.10	9.70	35.27–36.93	0.40
		Women	Male	30.52	7.08	29.91–31.13	37.95	8.96	37.19–38.72	0.92
			Female	33.60	9.29	32.8–34.39	40.04	11.04	39.09–40.99	0.63
	Aggressiveness	Men	Male	29.15	6.04	28.63–29.67	34.83	8.68	34.09–35.57	0.76
			Female	31.49	8.23	30.78–32.19	36.55	10.91	35.62–37.49	0.52
		Women	Male	30.66	7.42	30.03–31.3	37.36	10.67	36.44–38.27	0.73
			Female	33.68	9.12	32.9–34.46	40.29	11.71	39.29–41.3	0.63
	Trustworthiness	Men	Male	29.82	5.95	29.31–30.33	38.86	11.70	37.86–39.87	0.97
			Female	33.43	8.63	32.69–34.17	41.44	11.67	40.44–42.45	0.78
		Women	Male	30.26	6.91	29.66–30.85	33.27	7.60	32.62–33.92	0.41
			Female	33.94	9.23	33.15–34.74	36.71	9.52	35.89–37.53	0.29
Social perception	Attractiveness	Men	Male	4.71	2.09	4.54–4.89	4.37	1.95	4.2–4.54	0.17
			Female	4.94	2.25	4.75–5.13	4.64	2.12	4.46–4.82	0.14
		Women	Male	3.90	2.02	3.73–4.07	3.61	1.96	3.44–3.78	0.15
			Female	4.77	2.02	4.6–4.94	4.51	1.98	4.34–4.68	0.13
	Social Status	Men	Male	5.49	1.67	5.34–5.63	5.51	1.69	5.36–5.65	0.01
			Female	5.76	1.66	5.62–5.91	5.80	1.55	5.67–5.93	0.02
		Women	Male	5.36	1.80	5.21–5.52	5.18	1.98	5.01–5.35	0.10
			Female	5.51	1.75	5.36–5.66	5.39	1.79	5.23–5.54	0.07
	Aggressiveness	Men	Male	4.50	2.27	4.31–4.7	4.57	2.27	4.37–4.76	0.03
			Female	4.23	2.26	4.04–4.42	4.35	2.32	4.15–4.55	0.05
		Women	Male	5.01	2.37	4.8–5.21	5.06	2.34	4.86–5.26	0.02
			Female	4.39	2.22	4.2–4.58	4.29	2.19	4.11–4.48	0.04
	Trustworthiness	Men	Male	5.91	2.09	5.73–6.09	5.42	1.95	5.25–5.59	0.24
			Female	5.99	2.14	5.81–6.17	5.51	1.96	5.34–5.68	0.23
		Women	Male	4.79	1.81	4.63–4.94	4.80	1.83	4.64–4.95	0.01
			Female	5.71	1.98	5.54–5.88	5.61	1.97	5.45–5.78	0.05

## 4 Discussion

Both men and women perceived faces with gray hair as older and less attractive than faces without gray hair. Men (but not women) perceived faces with gray hair as less trustworthy than faces without gray hair. Social status and aggressiveness perceptions were similar irrespective of whether the faces had gray hair or not. These results support our predictions regarding perceived age and attractiveness and partially support our predictions about trustworthiness, social status and aggression.

Previous studies have found that people with gray hair are perceived as older. When evaluating images of men and women with varying levels of gray hair, people estimated both men and women as being older when they had more gray hair (Bulpitt et al., 2001; Gunn et al., 2009). Interestingly, women that dyed their hair (likely to non-gray natural colors) were perceived as appearing younger for their age compared to woman that did not dye their hair (Gunn et al., 2009). Our experimental results confirm these correlational studies, indicating a strong link between gray hair and perceived age. Given the relatively strong association between chronological age and gray hair (Keogh and Walsh, 1965; Panhard et al., 2012), gray hair is a relatively reliable index of age and may be used as an honest indicator of age (Jawor and Breitwisch, 2003).

Both male and females faces with gray hair were perceived as less attractive by men and women. Since faces with gray hair were perceived as older, these attractiveness judgments may be driven by perceived age of the faces. It is also possible that the attractiveness judgments were influenced by the relatively young age of the subjects or the modified stimuli (potentially appearing unnatural to the subjects). Many previous studies have found that older faces are considered less attractive than younger faces (Foos and Clark, 2011; Furnham et al., 2004; He et al., 2021; Kissler and Bäuml, 2000; Kwart et al., 2012; Wernick and Manaster, 1984). Women generally prefer romantic partners who are similar in age to themselves and men tend to prefer romantic partners who are within their reproductive years (Buunk et al., 2001; Kenrick and Keefe, 1992). Given that most of our women subjects were below 30 years old, the perceived ages of the male faces were closer to their own ages when the faces had non-gray hair versus gray hair. As such, the male faces with non-gray hair were closest in age to the women subjects, potentially explaining why they rated those faces as most attractive. Similarly, men perceived the female faces with non-gray hair as younger compared to the faces with gray hair, and therefore potentially perceived the faces with non-gray hair as more fertile (Berkowitz et al., 1990). It is also possible that faces with graying hair are perceived as less attractive because graying hair can be a sign of disease, such as Alzheimer’s disease (Mendelsohn and Larrick, 2020), Parkinson’s disease (Jucevičiūtė et al., 2019) or cardiovascular disease (Elfaramawy et al., 2018).

Gray hair had no impact on the perception of social status or aggression. Faces of men are perceived as reaching their peak dominance around 35 years old and then plateauing (Batres et al., 2015). We did not find that the male faces with non-gray hair were perceived as having lower social status, despite these faces being perceived as younger (around 30 years old) than the dominance plateau while the faces with gray hair were above the dominance plateau (around 36 years old). Similarly, even though male faces that are older are perceived as less physically dominant (Richardson et al., 2020), we did not find that male faces with gray hair (and thus perceived as being older) were considered less aggressive. These results suggest that traits aside from gray hair (such as wrinkles, baldness, facial hair, or masculinity; Batres et al., 2015; Bulpitt et al., 2001; Dixson and Vasey, 2012) are more important to assessments of social status and aggression.

Gray hair lowered the trustworthiness of faces when men assessed them. Previous studies have found that younger faces are considered more trustworthy than older faces but the gender of the faces and participants were either not considered or not found to have an effect (Pehlivanoglu et al., 2022; Zebrowitz et al., 2013). It is possible that men considered faces with gray hair less trustworthy because those faces were perceived as older. Other studies have found the opposite: older people are generally considered highly trustworthy (Cuddy and Fiske, 2002; Fiske, 1998). In contrast, women’s evaluation of trustworthiness was unaffected by gray hair. This suggests that other factors are more important in driving trustworthiness assessments in women, such as facial shape (Kleisner et al., 2013; Rezlescu et al., 2012; Siddique et al., 2022; Stirrat and Perrett, 2010; Todorov et al., 2008;), self-resemblance (DeBruine, 2002; Farmer et al., 2013), and typicality (Sofer et al., 2014). The finding that perceptions of trustworthiness was influenced by gray hair for men, but not women, might also be attributable to different sex-based priorities in social perceptions related to mate-choice (Buss, 1996). For example, from an evolutionary perspective, men tend to seek partners exhibiting youthful characteristics, while this tends not to be as strong a priority for women in mate-seeking (Buss and Schmitt, 2019). Youthful facial characteristics are generally perceived as more trustworthy (Zebrowitz, 2017), so it is possible that the increased perceptions of age due to gray hair detrimentally impacted perceptions of trustworthiness moreso for men than women, given the sex differences in ecological weight of youthful characteristics. Trustworthiness may also be especially important evolutionarily to men to ensure paternity (Buss and Schmitt, 1993; Apicella and Marlowe, 2004). Additional studies examining the links between gray hair, age, and trustworthiness as a function of sex would be valuable.

### 4.1 Limitations and future directions

We note several limitations in this study that could be explored in future research. First, the perceived age of the faces (likely Caucasian) with non-gray hair color used in this study was relatively young (around 30 years old). While Caucasian people usually begin to get gray hair in their mid-thirties (Keogh and Walsh, 1965), the percentage of gray hair is relatively low (most people below 40 years old have less than 20% gray hair; Kwon et al., 2012; Panhard et al., 2012). Due to the methodological approach of creating natural appearing gray hair while holding all other facial features constant, the gray hair that we simulated in this study was complete (100% gray hair). Therefore, it would be informative to evaluate how the degree of graying impacts perceived age and social perceptions. In addition, it is possible that subjects perceived the gray hair in these faces of relatively young people as prematurely gray, which can be associated with additional physiological and psychological conditions beyond natural aging processes (Thompson et al., 2019). Furthermore, it would be valuable to determine whether the faces of older people (those that are more likely to have higher levels of gray hair) would be evaluated differently. It would be particularly interesting to assess whether older men with gray hair are considered more attractive than those with less gray hair, as suggested by the vernacular term “silver fox.” Similarly, the perception of gray hair may vary based on the subjects’ age. Most of our subjects were relatively young and it is possible that older subjects would evaluate the faces with gray hair differently. Second, we modified hair color by simulating gray hair only on the head. As people age, the hair color of their eyebrows, eye lashes and facial hair can also turn gray (Senanayake and Wickramasinghe, 2016). We used male faces with minimal to no facial hair but some male faces did have some stubble. It would therefore be informative to assess how changes in hair color on these other facial regions impacts perceived age and social perceptions. Third, the face stimuli were likely Caucasian and evaluated by primarily Caucasian and Asian participants. Because the prevalence of gray hair varies by ethnic group (Panhard et al., 2012), further studies that examine the perception of gray hair by ethnic group could indicate ethnic differences in the perception of gray hair. And fourth, the face stimuli were assessed by subjects that were mostly likely from Western culture. Because social perceptions can vary across cultures (Tovée et al., 2006), it would be interesting to determine how the perception of gray hair varies by culture, especially in cultures that attribute high esteem to the elderly (North and Fiske, 2015).

Future studies could also consider modifying other aspects of this study. Other social perceptions (such as likability) may also be linked to gray hair and would be fruitful targets for future study. The faces in this study were displayed for 5 s each and it would be interesting to assess whether varying this viewing duration influences social perceptions. Previous work has demonstrated that people can assess the gender, age, and identity of faces in less than a second (Dobs et al., 2019) but viewing time can influence social perceptions (Rashidi et al., 2012). Because we modified only the hair color of the faces in our stimuli (and not other features often associated with aging such as gray eyebrows and wrinkles), it is possible that the face stimuli appeared unnatural or artificial to the subjects. Experiments with crossed designs that vary multiple traits (e.g., faces with gray hair, faces with wrinkles, and faces with both gray hair and wrinkles) could tease apart the importance of each feature. Future studies confirming that the face stimuli appeared natural to the subjects would also be valuable. Artificial faces, such as the faces of avatars, are not always perceived in the same way as real faces at the behavioral or neural level (Moser et al., 2007). Similarly, it is possible that subjects may have been biased to assess the faces with gray hair as older because of experimental expectations (e.g., if the subjects noted that the hair color was the only trait being modified and they assume that gray hair is usually associated with older age, they may have assessed the faces as older). This could explain why the faces with gray hair were only rated as 1.2× older than the faces with non-gray hair, rather than being rated as much older. In addition, hair turns gray in other species as well as they age, such as chimpanzees (Tapanes et al., 2020) and dogs (Pati et al., 2015). Future studies that examine whether gray hair in these non-human animals affects how conspecifics perceive them would be interesting.

By better understanding perceived age and social perceptions of faces, we can potentially use this knowledge to overcome implicit biases (Haselton et al., 2016). Given that faces with gray hair are perceived as older, it is possible that people with gray hair are discriminated against, as older individuals are often discriminated against in the workplace and other settings (reviewed in Marques et al., 2020). For example, people who appear older are less likely to be hired than younger-looking people (Kaufmann et al., 2016). Ageism is a form of conscious or unconscious discrimination against older people in which others alter their feelings, beliefs or behaviors because of an individual’s real or perceived age (Iversen et al., 2009; Levy and Banaji, 2002). This discrimination may result from an evolutionary mismatch such that behaviors that were adaptive in our ancestral environment are no longer adaptive today (Li et al., 2018). Being aware of biases is often among the first steps to overcoming them (Lee, 2017), suggesting that being aware of how gray hair color impacts age and social perceptions can be a first step to reducing biases against those with gray hair in contexts ranging from the workplace to other social situations. Interventions that aim to promote positive attitudes toward older people have some success in overcoming these biases (Sinclair et al., 2023). Among different types of interventions, age diversity workshops are among the most promising tools for reducing bias toward older people. For example, a workshop focused on reducing bias of older workers by awareness-raising and intergroup contact; biases toward older workers were reduced after the workshop, with positive effects persisting 6 months afterwards (Schloegel et al., 2016).

## 5 Conclusion

People frequently and rapidly make inferences about a person’s age, which can meaningfully inform social judgments and decision making in social interactions. Graying hair is an observable characteristic that can convey age information, and indirectly inform other relevant social perceptions. The current work conducted an experiment that independently manipulated hair color while assessing perceptions of age and related social evaluations. The results demonstrated that gray hair increased perceptions of age, decreased perceptions of attractiveness, decreased perceptions of trustworthiness (rated by men, but not women), and did not impact perceptions of social status or aggressiveness. These results suggest that gray hair can independently be used as an indicator of some important social evaluations (age, attractiveness, trustworthiness), while others (social status, aggression) may be better informed by other visible characteristics. The current work highlights the role of observable characteristics in informing social inferences, the utility of experimental approaches toward separating the unique contributions of independent visible signals of social states and traits, and potentially informs approaches to minimize social biases related to aging.

## Data Availability

The original contributions presented in the study are included in the article/Supplementary material, further inquiries can be directed to the corresponding author.
