# Supplementary material for: Gray hair influences perceived age and social perceptions
Source: Front Psychol. 2025 May 7;16:1541836. doi: 10.3389/fpsyg.2025.1541836 (PMC12094188; doi:10.3389/fpsyg.2025.1541836)
Supplement: Supplementary file 1 [file Supplementary_file_1.docx]

Table S1. The effect of stimuli hair color, stimuli gender, and subject gender on perceived age in each experiment with the dataset excluding short and long reaction times. F values with p-values in parentheses are displayed. Statistically significant results are indicated with an asterisk.

|  | **Attractiveness: Age** | **Social Status: Age** | **Aggression: Age** | **Trustworthiness: Age** |
| --- | --- | --- | --- | --- |
| **Stimuli Hair Color** | 62.14 (<0.0001)* | 17.07 (0.0003)* | 17.26 (0.0003)* | 17.67 (0.0002)* |
| **Stimuli Gender** | 39.15 (<0.0001)* | 118.18 (<0.0001)* | 99.67 (<0.0001)* | 149.53 (<0.0001)* |
| **Subject Gender** | 0.49 (0.49) | 2.34 (0.14) | 1.33 (0.26) | 1.51 (0.23) |
| **Stimuli Gender x Stimuli Hair Color** | 8.33 (0.0074)* | 0.90 (0.35) | 0.010 (0.93) | 3.37 (0.077) |
| **Stimuli Gender x Subject Gender** | 4.90 (0.035)* | 0.00 (0.97) | 1.62 (0.21) | 0.080 (0.79) |
| **Stimuli Hair Color x Subject Gender** | 0.52 (0.48) | 42.33 (<0.0001)* | 9.21 (0.0052)* | 119.44 (<0.0001)* |
| **Stimuli Hair Color x Stimuli Gender x Subject Gender** | 5.80 (0.023)* | 2.22 (0.15) | 0.010 (0.94) | 0.81 (0.38) |
| **Perception of Ageing** | 0.27 (0.61) | 3.05 (0.093) | 0.19 (0.67) | 0.24 (0.63) |
| **Perception of Ageing x Stimuli Hair Color** | 11.39 (0.0007)* | 0.42 (0.52) | 0.29 (0.59) | 0.22 (0.64) |
| **Subject Age** | 0.040 (0.84) | 0.68 (0.42) | 0.00 (0.99) | 0.050 (0.83) |

Table S2. The effect of stimuli hair color, stimuli gender, and subject gender on social perceptions with the dataset excluding short and long reaction times. For the overall model, F values with p-values in parentheses are displayed; for the comparisons, t values with p-values in parentheses are displayed. Statistically significant results are indicated with an asterisk.

|  | | | **Attractiveness** | **Social Status** | **Aggression** | **Trustworthiness** |
| --- | --- | --- | --- | --- | --- | --- |
| **Overall Model** | | |  |  |  |  |
| Stimuli Hair Color | | | 4.92 (0.035)* | 0.98 (0.33) | 2.22 (0.15) | 2.36 (0.14) |
| Stimuli Gender | | | 73.41 (<0.0001)* | 20.23 (0.0001)* | 53.6 (<0.0001)* | 65.74 (<0.0001)* |
| Subject Gender | | | 0.41 (0.53) | 0.15 (0.70) | 0.46 (0.51) | 2.09 (0.16) |
| Stimuli Hair Color x Stimuli Gender | | | 0.03 (0.86) | 0.38 (0.54) | 0.15 (0.70) | 0.33 (0.57) |
| Stimuli Gender x Subject Gender | | | 34.35 (<0.0001)* | 0.54 (0.47) | 17.86 (0.00020)* | 48.98 (<0.0001)* |
| Stimuli Hair Color x Subject Gender | | | 0.48 (0.50) | 2.85 (0.10) | 0.61 (0.44) | 12.54 (0.0014)* |
| Stimuli Hair Color x Stimuli Gender x Subject Gender | | | 0.00 (0.95) | 0.18 (0.68) | 0.87 (0.36) | 0.090 (0.76) |
| Perception of Ageing | | | 3.58 (0.070) | 4.70 (0.040)* | 1.54 (0.23) | 0.030 (0.87) |
| Perception of Ageing x Stimuli Hair Color | | | 1.39 (0.24) | 0.56 (0.45) | 2.51 (0.11) | 4.62 (0.032) |
| Subject Age | | | 0.21 (0.65) | 5.82 (0.023)* | 0.00 (0.99) | 0.070 (0.80) |
|  | | |  |  |  |  |
| **Comparisons** | | |  |  |  |  |
| **Subject Gender** | **Stimuli Gender** | **Stimuli Hair Color** |  |  |  |  |
| **Men** | Men | Natural vs. gray | 3.40 (0.0021)* | 0.43 (0.67) | 0.39 (0.70) | 3.40 (0.0021)* |
|  | Women | Natural vs. gray | 3.29 (0.0027)* | 0.63 (0.54) | 0.92 (0.37) | 3.58 (0.0013)* |
| **Women** | Men | Natural vs. gray | 2.70 (0.012)* | 1.68 (0.10) | 0.53 (0.60) | 0.46 (0.65) |
|  | Women | Natural vs. gray | 2.52 (0.018)* | 1.47 (0.15) | 0.79 (0.43) | 0.42 (0.67) |
